# Pathogenic mechanisms and treatment advances in proliferative vitreoretinopathy: A review

**DOI:** 10.1097/MD.0000000000044804

**Published:** 2025-09-26

**Authors:** Jinhui Dai, Xiaorong Zhou, Haixia Bai, Meiyan Zeng, Qinghua Peng, Quanlong Wu

**Affiliations:** a The First Clinical College of Traditional Chinese Medicine, Hunan University of Traditional Chinese Medicine, Changsha, China; b The First Affiliated Hospital of Hunan University of Chinese Medicine, Changsha, China; c Hunan Province Traditional Chinese Medicine Prevention and Treatment of Eye, Ear, Nose and Throat Diseases and Visual Function Protection Engineering Technology Research Center, Changsha, China.

**Keywords:** cells, inflammatory mediators, pathways, proliferative vitreoretinopathy

## Abstract

Proliferative vitreoretinopathy (PVR) is the most common cause of repair failure in rhegmatogenous retinal detachment and a severe complication of posterior segment penetrating injuries. The pathogenesis of PVR is complex, and there is currently no ideal therapeutic drug in clinical practice. Surgical intervention involves the removal of proliferative membranes to relieve traction on the retina, but it carries a certain postoperative recurrence rate. This review provides an overview of the pathogenesis and treatment strategies for PVR.

## 1. Introduction

Proliferative vitreoretinopathy (PVR) is fundamentally an intraocular fibrotic disease.^[1,2]^ The occurrence and progression of PVR result from the combined effects of various factors, leading to damage to the blood-retinal barrier. The hallmark of PVR is the formation of contractile fibrocellular proliferative membranes within the vitreous cavity, on the surface of the retina, and beneath the retina.^[3]^ These membranes exert traction on the retina, causing retinal detachment (RD) and ultimately leading to visual impairment or even blindness.^[4]^ Despite the increasing refinement of intraocular surgical techniques and the significant improvement in surgical success rates, vitreoretinal surgery aims to restore the anatomical structure of the retina through mechanical means. However, it does not restore retinal function, and the surgery may stimulate cellular proliferation and migration within the eye, potentially leading to PVR recurrence.

Due to the complex nature of PVR formation, which is similar to abnormal wound healing responses, various cell types, including inflammatory cells, retinal cells, and retinal pigment epithelium (RPE) cells, along with multiple cytokines, are involved.^[5]^ Therefore, research on cellular molecular targets and related pathways involved in PVR formation is crucial. This review provides a comprehensive overview of the pathogenic mechanisms and treatment strategies for PVR, offering new insights for further research on PVR prevention and treatment.

Currently, it is widely accepted that the occurrence of epithelial-mesenchymal transition (EMT) in RPE cells is a key factor in the pathogenesis of PVR.^[6]^ Studies indicate that RPE cells play a central role in the pathological process of PVR.^[7,8]^ Following rhegmatogenous RD or posterior segment penetrating injury, the tight connections between RPE cells are damaged. Subsequently, RPE cells detach from the Bruch membrane and disperse into the vitreous cavity through retinal breaks. Concurrently, stimuli such as inflammation, hypoxia, and damage to the blood-retinal outer barrier lead to the influx of circulating cytokines and macrophages into the vitreous cavity.^[9]^ These factors, in conjunction with preexisting cytokines within the vitreous cavity, collectively act on RPE cells, triggering EMT.^[10,11]^ This process results in RPE cell migration, proliferation, transformation into fibroblasts and myofibroblasts (Mfbs), synthesis, and secretion of extracellular matrix (ECM), ultimately forming a fibrotic proliferative membrane and causing tractional retinal detachment (TRD).^[12]^

**Figure 1.**
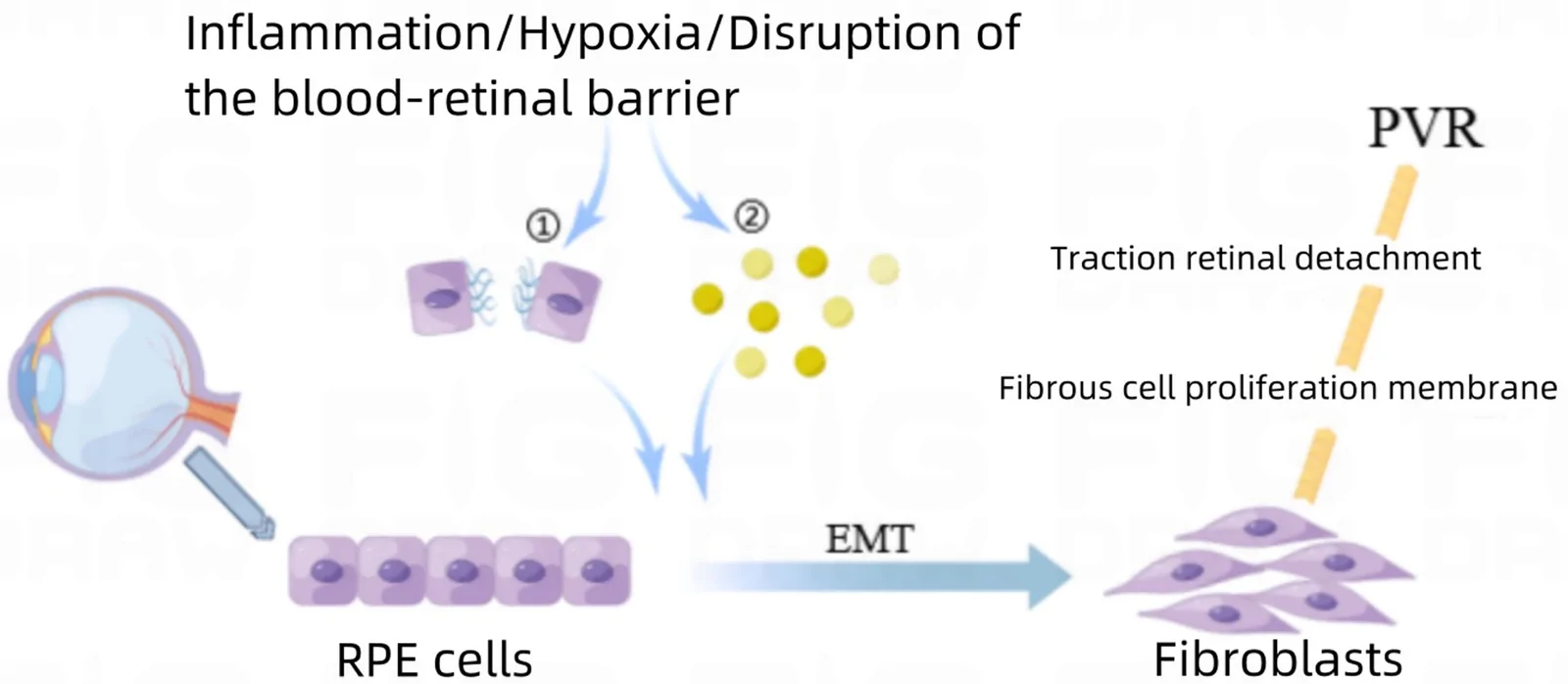
Schematic representation of the pathogenic mechanism of PVR. Note: ① Breakdown of cell junction complexes; ② Action of cytokines. EMT = epithelial-mesenchymal transition, PVR = proliferative vitreoretinopathy, RPE = retinal pigment epithelial.

EMT can be activated in pathological conditions such as inflammation, wound healing, and tumorigenesis, enabling epithelial cells to acquire enhanced migratory capabilities and increase the production of ECM components. In the normal vitreous and retina, Mfbs are absent. Through type 2 EMT, RPE cells proliferate and gradually lose their epithelial morphology, transforming into Mfb-like cells.^[13,14]^ In the pathogenesis of PVR, RPE cells undergo type 2 EMT, transforming into Mfbs (see Fig. 1).^[9–14]^

Abundant histopathological data reveal that the primary components of PVR proliferative membranes are fibrous tissue, primarily composed of RPE cells, macrophages, glial cells, fibroblasts, and various ECM elements.^[11,15]^ Laboratory reports indicate the presence of soluble apoptotic factors, adhesion factors, transforming growth factor-β (TGF-β), platelet-derived growth factor (PDGF), interleukins (ILs), tumor necrosis factor, and various growth factors and cytokines excessively expressed in the vitreous or subretinal fluid of PVR patients.^[16–19]^ This suggests that the abnormal expression of these inflammatory mediators drives PVR, ultimately leading to the formation and subsequent contraction of preretinal membranes.^[20–22]^

## 2. RPE cells

RPE cells play a central role in PVR.^[7,8]^ Situated as a monolayer of pigment-containing cells between the neuroepithelial layer of the retina and the choroid, RPE cells form the blood-retinal outer barrier through tight junction complexes, preventing free movement of water and ions. Human RPE cells reach terminal differentiation at 4 to 6 weeks of gestation and subsequently remain in a mitotically quiescent state. Under normal circumstances, RPE cells do not proliferate or undergo transdifferentiation. However, when subjected to retinal breaks or trauma disrupting the physiological hierarchy of vitreoretinal tissue, RPE cells become exposed to various growth factors and cytokines produced by activated immune cells. This exposure results in the breakdown of RPE cell junction complexes, leading to the detachment of activated RPE cells from the Bruch membrane.^[23]^ Subsequently, activated RPE cells migrate, proliferate, synthesize collagen, and other ECM components. When RPE cells come into contact with cell cycle-promoting factors, EMT occurs,^[10,11]^ transforming them into Mfb-like cells. This transformation contributes to the formation of contractile fibrotic membranes.^[24]^ Similar to wound healing responses, these membranes can adhere to the retina and contract, further causing RD.

## 3. Neuroglial cells

In PVR, neuroglial cells, commonly found within the membranes surrounding the retina, undergo proliferation in response to retinal damage. Müller cells represent a specific type of neuroglial cell. Glial fibrillary acidic protein is considered a sensitive biomarker for reactive Müller glial proliferation, showing a positive correlation with the severity of PVR.^[25]^ Müller cells, when triggered by various inflammatory cytokines, release additional cytokines and growth factors into the local retinal environment, promoting the proliferation of neuroglial cells and tissue damage.^[26]^ These cells move outside the retina through ruptures or gaps in the internal limiting membrane, where they can proliferate, contract, and synthesize collagen, providing focal points for the proliferation and contraction of other cells. Müller cells serve as a rich source of cytokines, contributing to processes such as cell proliferation, migration, inflammation, and fibrosis. Pathological hypertrophy and proliferation of Müller cells can lead to retinal shortening and deformation.^[11]^

## 4. Macrophages

Macrophages play a crucial role in the early stages of PVR formation, acting as significant stimulants for PVR. They mediate inflammatory reactions, which are necessary for cell proliferation.^[27]^ Firstly, they participate in the formation of PVR membranes on the retinal surface. Secondly, similar to Müller neuroglial cells, they serve as a rich source of cytokines, mediating various cellular processes and releasing cytokines.^[28]^ They can produce growth factors such as PDGF, fibroblast growth factor, TGF-β, ILs, among others, participating in the regulation of injury healing. Thirdly, macrophages cannot proliferate or contract, but they can induce the transformation of vascular adventitial cells into fibroblasts or Mfbs. These cells can synthesize fibrous binding proteins and likely play a role in the pathogenesis of PVR.

## 5. Transforming growth factor-β

TGF-β is a multifunctional cytokine isoform that regulates cell differentiation, migration, apoptosis, immune function, and ECM synthesis. It is expressed at high concentrations in the vitreous of PVR patients.^[29]^ In the eye, TGF-β is believed to maintain immune privilege by inhibiting antigen-driven T-cell activation and proliferation. However, in the context of PVR, TGF-β stimulates activity and is present in high concentrations in the vitreous, proportional to the degree of fibrosis.^[30]^ It induces EMT and the production of type I collagen and ECM in RPE cells through intracellular signaling cascades.^[11]^ TGF-β1 is stored or activated in photoreceptors, TGF-β2 is observed in the ciliary artery and photoreceptor outer segments,^[31]^ and TGF-β3 is expressed in isolated single cells in the choroid and retina.^[26]^ TGF-β participates in PVR retinal contraction, activates macrophages and RPE cells, and generates TGF-β2. The severity of fibrosis is directly proportional to TGF-β2 levels. TGF-β3 subtype is significantly upregulated in the vitreous of eyes with RD compared to eyes with preretinal membranes. TGF-β3 and IL-8 increase simultaneously, indicating simultaneous apoptosis and biologically active processes involving ECM synthesis in cases involving more retinal quadrants.^[32–35]^ In rabbit PVR models, TGF-β1 and TGF-β2 are significantly upregulated in the aqueous humor and vitreous.^[36]^ Progression of PVR is associated with TGF-β-induced EMT in RPE cells.^[37]^ TGF-β1 plays a crucial role in EMT in RPE cells by activating the TGF-β-activated kinase 1.^[38]^ TGF-β activates various downstream pathways, including the Smad-dependent pathway regulating target gene expression. It induces the expression of mesenchymal markers such as smooth muscle α-actin and vimentin, while reducing the expression of epithelial markers such as ZO-1.^[39–42]^ Tissue fibrosis and contraction heavily depend on the TGF-β/Smad pathway. Thus, blocking Smad signaling can effectively suppress the fibrogenic response of EMT and the transformation from fibroblasts to Mfbs.^[35,43]^

## 6. TGF-β/Smads signaling pathway

TGF-β is a multifunctional polypeptide growth factor and a potent EMT chemoattractant. By binding to specific receptors and activating a signaling pathway, TGF-β regulates the EMT process and plays a crucial role in various fibrotic-related diseases, serving as a significant inducer of PVR. TGF-β induces RPE cells to transform into fibroblasts and Mfbs, promoting cell proliferation, migration, excessive ECM generation, participating in fibrous membrane formation, and expressing α-smooth muscle actin in stress fibers, increasing fibrous membrane contractility, ultimately leading to TRD.^[44]^ These processes play a key role in the pathogenesis of PVR. TGF-β’s effects span RPE cell proliferation, differentiation, migration, adhesion, and the contraction process of fibrous proliferative membranes.^[45]^ Studies indicate that bortezomib, by blocking TGF-β1 signaling, reduces the transformation of RPE cells into Mfbs.^[46]^ TGF-β2 is highly expressed in the vitreous of PVR patients, correlating with the degree of fibrosis,^[47]^ suggesting an essential role for TGF-β in the EMT process of RPE cells. Research confirms that the classical TGF-β/Smads signaling pathway is closely associated with the EMT process in RPE cells.

**Figure 2.**
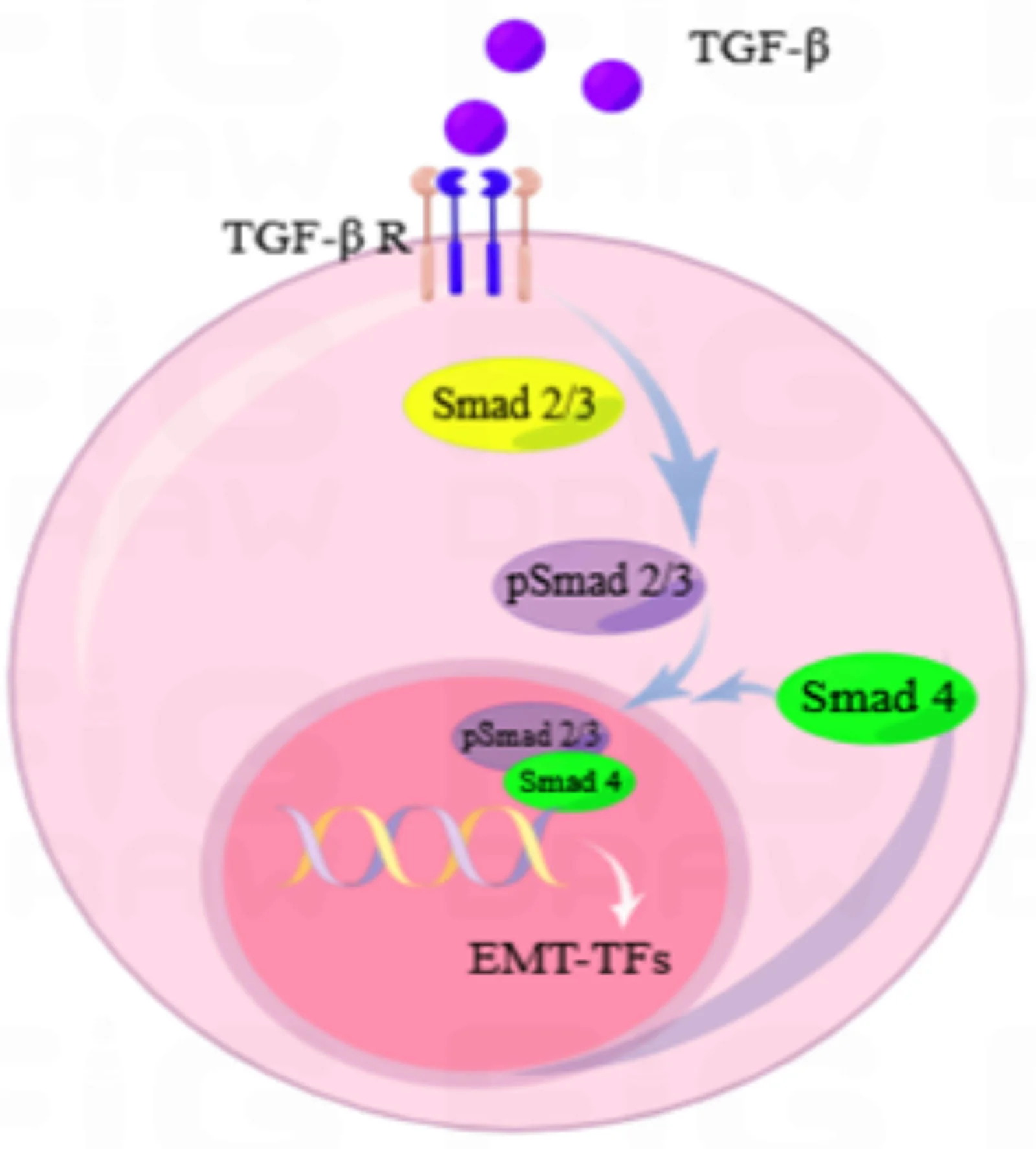
Schematic representation of the TGF-β/Smads signalling pathway in mediating EMT. EMT = epithelial-mesenchymal transition, TGF-β = transforming growth factor-β.

TGF-β activates the Smad signaling pathway by binding to type II TGF-β receptors (TGF-βR2) on the cell surface. Subsequently, it recruits type I TGF-β receptors (TGF-βR1) and initiates downstream signal transduction. Smad2 and Smad3 are recruited and phosphorylated, forming a complex with Smad 4, which translocates to the cell nucleus. This complex regulates transcription of target genes, promoting the expression of fibrotic genes and facilitating EMT (see Fig. 2).^[39–43]^ Lee et al found that rhCD82 inhibits TGF-β1-induced EMT in RPE by blocking the Smad-dependent pathway.^[48]^ Additionally, blocking the TGF-β and FGF/MAPK pathways has been shown to significantly enhance the differentiation efficiency of RPE cells derived from induced pluripotent stem cells (hiPSCs).^[49]^ Since the TGF-β signaling pathway plays a crucial role in mediating EMT and ECM generation, targeting the TGF-β signaling pathway could be one of the strategies to inhibit PVR.

## 7. Platelet-derived growth factor

In eyes afflicted with PVR, heightened expression of PDGF and its receptors is observed in both RPE cells and Müller cells. Research indicates that PDGFα receptor (PDGFR-α) is activated in eyes affected by PVR.^[50]^ PDGF participates in various cell chemotaxis and proliferation processes related to PVR, stimulating collagen production, promoting ECM formation, and inducing cell contraction through close adhesion.^[51]^ It stands as one of the factors contributing to the formation and progression of PVR. Studies on choroidal neovascularization suggest that blocking PDGFR signaling weakens laser-induced neovascularization and subretinal fibrosis in PVR patients. Elevated expression of PDGF and PDGFR detected in the vitreous of PVR patients confirms their involvement in the pathogenesis.^[11,52]^ Additionally, PDGFR is found to play a crucial role in the contraction of retinal pigment epithelial cells stimulated by the vitreous.^[53]^ Inhibition of PDGFR activation is identified as a key element in preventing and treating PVR.^[54]^ Among the 3 subtypes of PDGFR, PDGF-C can activate PDGFRα and PDGFRαβ.^[55,56]^ Saffron extract has been demonstrated as an effective inhibitor of PDGF-BB-induced proliferation and migration of RPE cells.^[57]^ Besides PDGF, non-PDGF factors such as epidermal growth factor, basic fibroblast growth factor, hepatocyte growth factor, and indirectly activate PDGFR, sustaining signals for cell survival, proliferation, and contraction involved in PVR development.^[20,21]^

## 8. Interleukins

ILs are secreted by various cells, stimulating cellular responses. Apart from their role in activating and regulating immune cells, ILs serve as crucial signals for abnormal proliferation, differentiation, and enhanced inflammation in T and B lymphocytes.^[58,59]^ Recent studies suggest a connection between ILs and the occurrence and development of PVR.^[17,18]^ IL-6, a multifunctional cytokine, regulates acute-phase inflammatory reactions, hematopoiesis, and inflammation.^[60]^ IL-6 is produced by various cells, including RPE cells, endothelial cells, fibroblasts, neutrophils, monocytes, and macrophages, in response to inflammatory signals.^[61]^ IL-6 exhibits both pro-inflammatory and anti-inflammatory effects in the eyes and elsewhere.^[62–64]^ IL-6 is identified as a key mediator in the complex process of postoperative PVR development.^[65]^ IL-6 levels correlate with the severity of PVR, the expression of matrix metalloproteinases, especially MMP2 and tissue inhibitors of metalloproteinases.^[65–71]^ IL-6 stimulates corneal epithelial cells and stromal fibroblasts, along with macrophages, to produce fibrogenic vascular endothelial growth factor.^[65]^ Similar to most inflammatory cytokines, IL-6 is present in high concentrations in subretinal fluid during RD and rhegmatogenous RD repair processes.^[72,73]^ Its presence is associated with the subsequent development of postoperative PVR,^[66]^ particularly elevated in the vitreous of early PVR patients.^[74,75]^ IL-8 is considered a pro-inflammatory chemokine with various chemoattractant properties. IL-8 plays a crucial role in eye inflammation through cell proliferation, differentiation, and activation,^[76]^ besides acting as a major chemoattractant for neutrophils and T lymphocytes. IL-8 is mainly secreted by RPE cells, macrophages, and endothelial cells.^[77–80]^ Studies have reported elevated levels of IL-8 in PVR patients, suggesting its potential role in promoting the progression of PVR.^[81–83]^ IL-1α/β (IL-1α and IL-1β) are 2 major subtypes of IL-1. While IL-1α is biologically active, IL-1β is activated by inflammasomes.^[84]^ Once activated, both subtypes function similarly to potent pro-inflammatory cytokines, influencing cell proliferation, differentiation, and regulating the functions of immune-activated cells, initiating and enhancing immune and inflammatory responses. In animal models, IL-1 induces a non-proliferative response and produces PVR membranes in mouse eyes with existing retinal perforations.^[85]^ The early response to RD involves the secretion of IL-1β by macrophages, which may promote photoreceptor death through NLRP3 inflammasome activation and stimulate RPE cells to upregulate inflammatory cytokines, including IL-6.^[86]^ IL-1 receptor antagonist single-nucleotide polymorphism is associated with the risk of PVR, supporting the role of IL-1 in the pathogenesis of PVR.^[87]^

## 9. NF-κB/NLRP3/IL-1β inflammatory axis

The NLRP3 inflammasome is defined as a protein complex composed of nucleotide-binding oligomerization domain-like receptors, capable of activating inflammatory enzymes and ultimately leading to the production of IL-1β.^[88]^ Activation of the NLRP3 inflammasome occurs through both canonical and noncanonical pathways. Both pathways respond to Toll-like receptor activation or certain cytokine receptors, which promote the formation of pro-IL-1β and pro-IL-18 forms and the transcription of NLRP3. All these processes are orchestrated through NF-κB and translocated to the cell nucleus.^[89]^ IL-1β is a secreted protein, and its activation and release require a crucial mediator, namely NLRP3, which cleaves pro-IL-1β into its mature form, subsequently secreting it into the local environment.^[90]^ Both NLRP3 and IL-1β play roles in the pathogenesis of numerous inflammatory diseases. However, the involvement of IL-1β in fibrosis is complex, primarily due to its relationship with inflammatory cytokines and TGF-β. According to Luo et al, transient exposure to IL-1β inhibits the action of TGF-β via the NF-κB pathway, thereby suppressing the Smad pathway responsible for the transcription of TGF-β encoding genes.^[91]^ Given that TGF-β plays a crucial role in various fibrosis-related diseases and is a significant factor inducing PVR, studies indicate that blocking TGF-β1 signaling reduces the transformation of RPE cells into Mfb-like cells.^[46]^ The concentration of TGF-β2 in the vitreous of PVR patients correlates with the degree of fibrosis,^[47]^ suggesting its important role in the EMT process of RPE cells. Hence, the NF-κB/NLRP3/IL-1β inflammatory axis is closely associated with RPE cells.

**Figure 3.**
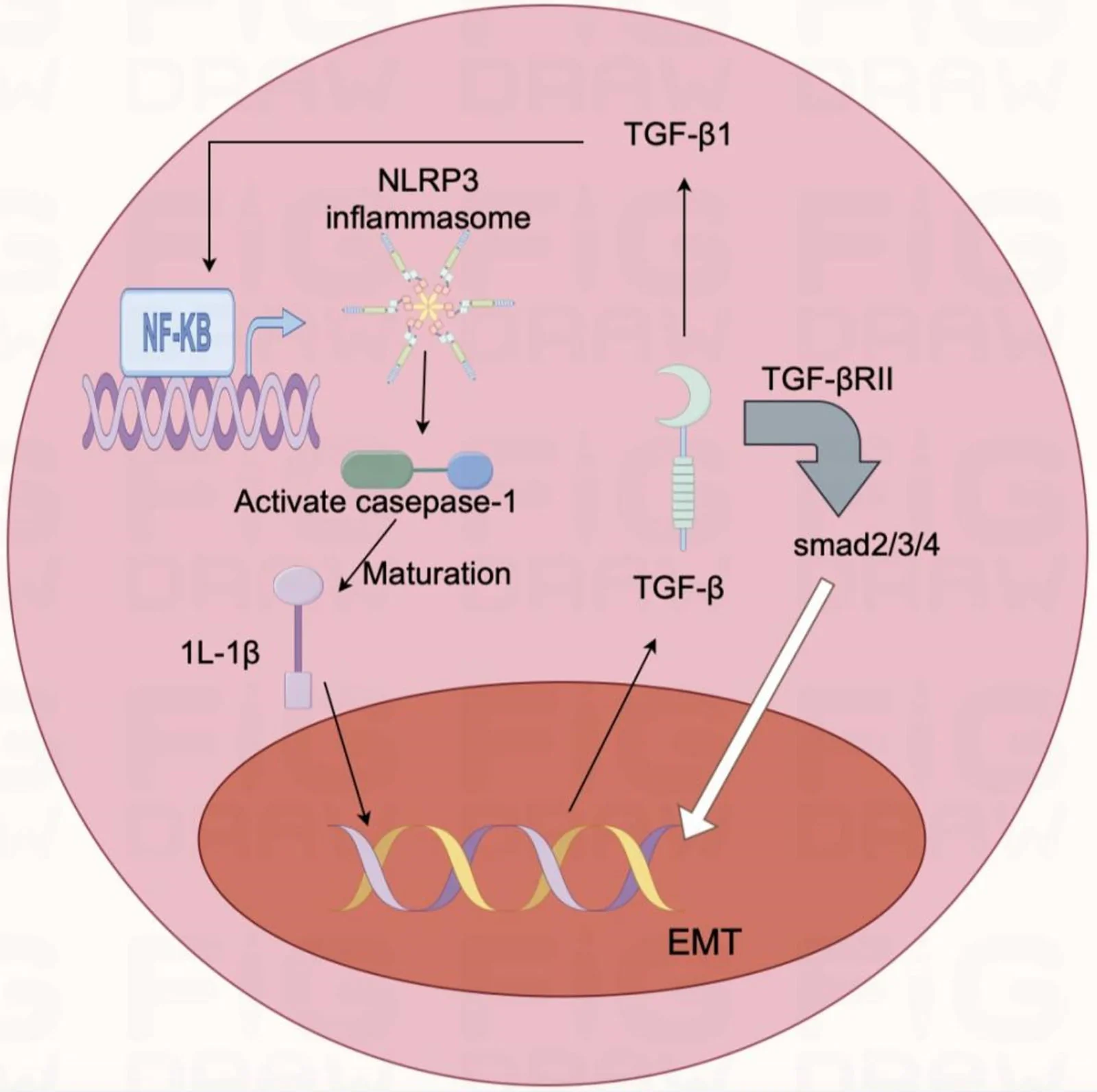
Schematic representation of the NF-κB/NLRP3/IL-1β inflammatory axis mediating EMT. EMT = epithelial-mesenchymal transition, IL = interleukin, TGF-β = transforming growth factor-β.

The activation of the NLRP3 inflammasome complex has the function of activating IL-1β, and IL-1β leads to increased TGF-β expression. Specifically, the NLRP3 inflammasome complex triggers EMT by inducing TGF-β derived from the production of IL-1β.^[92]^ In this scenario, EMT occurs through IL-1β signaling, as it binds to its receptor, triggering a series of signals that promote TGF-β transcription via MyD88 and c-JUN transcription factors. TGF-β can act in an autocrine and paracrine manner by binding to TGF-βRII, ultimately leading to the phosphorylation of Smad2/3 and their association with Smad4 as transcription factors for EMT-related genes.^[92,93]^ The NLRP3 inflammasome complex activates after 2 signals. The first signal (priming phase) promotes the transcription of inflammasome-related proteins (NLRP3, Pro IL-1β, and Pro IL-18) through NF-κB. The second signal (activation phase) facilitates the oligomerization of the complex itself, promoting the maturation of the cytokines IL-1β and IL-18 through caspase-1. Subsequently, the mature form of IL-1β reaches its receptor through autocrine, paracrine, or endocrine pathways, promoting the transcription of TGF-β genes. Secreted TGF-β binds to its receptor, triggering the Smad-dependent pathway, thereby promoting the EMT process (see Fig. 3).^[91–93]^

## 10. Treatment strategies

### 10.1. Surgical intervention

The primary approach for PVR treatment involves surgical intervention. With advancements in surgical techniques, the success rates of PVR surgeries have significantly improved. The main surgical goal is to alleviate traction and reconnect the detached retina. However, in cases of severe PVR, additional procedures may be required to mitigate traction, reconnect the retina, and prevent re-detachment.^[94]^ Over the past few decades, both anatomical and functional success rates have been less than satisfactory. Therefore, novel treatment strategies are needed to prevent or impede the progression of PVR.

### 10.2. Pharmacological treatment

Steroids have been investigated as a potential therapeutic option. In the context of PVR, initial use involved intravenous injection of triamcinolone acetonide to assist in visualizing and removing epiretinal membranes during RD vitrectomy.^[95]^ Zhao et al explored the impact of sustained-release dexamethasone on PVR cases.^[96]^ In PVR patients, the implantation of sustained-release dexamethasone during vitrectomy and silicone oil removal procedures reduced cystoid macular edema but did not enhance surgical success rates.^[97]^

Based on the growth factor and cytokine hypothesis, inhibitors with activity against the growth factor pathway are considered promising therapeutic targets for PVR treatment. Decorin, a core proteoglycan, is a natural TGF dual inhibitor. In a rabbit PVR model, it was found to reduce fibrosis and TRD.^[98]^ Cinar et al^[99]^ discovered that imatinib, by downregulating the expression of transcription factors Twist, Snail, and ZEB1 induced by TGF-β2, significantly inhibited TGF-β2-induced cell migration. Imatinib also suppressed RPE cell proliferation induced by TGF-β2 by regulating the cell cycle and promoting apoptosis, thereby inhibiting RPE cell proliferation and the EMT process. Rho kinase (ROCK), a key downstream mediator of TGF-β, has been demonstrated to effectively halt PVR progression in a rabbit model.^[100]^ AG1295, a specific inhibitor of PDGFR, was found to block PVR development by selectively inhibiting tyrosine kinase phosphorylation of PDGF-β receptor and its downstream signaling pathways, showing no apparent cytotoxicity. However, its therapeutic effect was evident only in the early stages of PVR.^[101]^ On the other hand, AG1296, primarily targeting PDGF-α receptor, exhibited inhibitory effects during both the proliferative and contractile stages of PVR.^[102]^ Recent studies have confirmed that PDGFR-α mRNA inhibitors can inhibit the proliferation of human retinal pigment epithelial cells, providing a new direction for PVR treatment.^[103]^

Methotrexate (MTX), a folate antagonist, inhibits cell proliferation and inflammatory pathways by increasing extracellular adenosine release at low doses. Adenosine can inhibit macrophage activation, leukocyte recruitment, and neutrophil adhesion.^[104]^ A retrospective study evaluating PVR and recurrent retinal tractional detachment patients showed lower PVR incidence in patients receiving intravitreal MTX during vitrectomy.^[105]^ Research also indicates that MTX treatment inhibits inflammation typically associated with silicone oil filling.^[106]^

## 11. Conclusions

In summary, the pathogenesis of PVR is extremely complex, involving multiple cells and inflammatory mediators, with RPE cells playing a major role; Müller cells and macrophages are the main sources of cytokines, and Müller cells contribute to cell proliferation, migration, and fibrosis, thereby promoting the progression of PVR. Macrophages are important stimuli for PVR, and their mediated inflammatory response is a crucial condition for cell proliferation. The EMT generated by RPE cells plays a crucial role in PVR, and the TGF-β/Smads signaling pathway guided by TGF-β and the NF-κB/NLRP3/IL-1β inflammatory axis can trigger EMT, leading to the development of PVR. In addition, PDGF is involved in various cell chemotaxis and proliferation of PVR, promoting ECM formation; IL-6, IL-8, and IL-1 all play important roles in the inflammatory response of the eye, promoting cell proliferation and playing a crucial role in the progression of PVR.

Despite some progress in PVR research, its treatment and prevention remain challenging in clinical practice. Surgical intervention remains the only currently effective treatment method, yet the recurrence rate posttreatment is high. Understanding the mechanisms underlying PVR formation and developing effective alternative treatments are pressing issues that need urgent resolution. This review provides an overview of the pathogenic mechanisms and treatment progress of PVR, aiming to offer new perspectives for understanding the occurrence, treatment, and prevention of PVR.

## Acknowledgments

We would like to thank figdraw.com for illustration drawing.

## Author contributions

**Data curation:** Jinhui Dai, Haixia Bai.

**Formal analysis:** Jinhui Dai, Xiaorong Zhou.

**Investigation:** Qinghua Peng.

**Supervision:** Meiyan Zeng.

**Visualization:** Quanlong Wu.

**Writing – original draft:** Jinhui Dai, Xiaorong Zhou, Haixia Bai, Meiyan Zeng, Qinghua Peng, Quanlong Wu.

**Writing – review & editing:** Jinhui Dai, Xiaorong Zhou, Haixia Bai, Meiyan Zeng, Qinghua Peng, Quanlong Wu.
